# Living Donation and Pre-Emptive Transplantation Are More Important Than HLA Matching in Pediatric Kidney Transplantation: Results From a 33-Year Comparative OPTN Study

**DOI:** 10.3389/ti.2025.15064

**Published:** 2025-11-12

**Authors:** Alicia Paessler, Ioannis D. Kostakis, Ioannis Loukopoulos, Zainab Arslan, Nicos Kessaris, Jelena Stojanovic

**Affiliations:** 1 Department of Nephrology, Great Ormond Street Hospital for Children NHS Foundation Trust, London, United Kingdom; 2 Department of Transplantation, Royal Free NHS Foundation Trust, London, United Kingdom; 3 Department of Transplantation, Guy’s and St Thomas’ Hospital NHS Foundation Trust, London, United Kingdom; 4 Evelina London Children’s Hospital, Guy’s and St Thomas’ Hospital NHS Foundation Trust, London, United Kingdom; 5 UCL Institute of Child Health, London, United Kingdom

**Keywords:** pediatric, kidney transplant, HLA mismatch, registry, survival analysis

## Abstract

Poorly HLA matched transplants have poorer long-term outcomes, however it is unclear whether living donation or pre-emptive transplantation can counteract the effects of HLA mismatches. We reviewed the long-term outcomes of paediatric kidney transplants with different HLA matches and aimed to identify other factors which may contribute significantly to long-term outcomes. We conducted a retrospective registry analysis of all pediatric kidney transplants from 1987–2020 in the USA from the OPTN Registry. These were analysed by HLA mismatches and compared by pre-transplant dialysis status and donor type. 21,500 patients were included for analysis. Overall, patients with unfavourable HLA matches had higher rates of delayed allograft function and lower allograft survival. However, patients with unfavourable HLA matched transplants from living donors had better allograft survival than patients with favourable HLA matched transplants from deceased donors (79% at 5 years vs. 71%, p < 0⋅01). Patients with pre-emptive unfavourable HLA matched transplants had better allograft and patient survival than patients with non-pre-emptive favourable HLA matched transplants (83% at 5 years vs. 78%, p = 0⋅02% and 98% vs. 96%, p < 0⋅01 respectively). In conclusion, living donation and pre-emptive transplantation have a more significant impact on clinical outcomes and lead to better allograft and patient survival than HLA matching.

## Graphical Abstract

**Figure:**
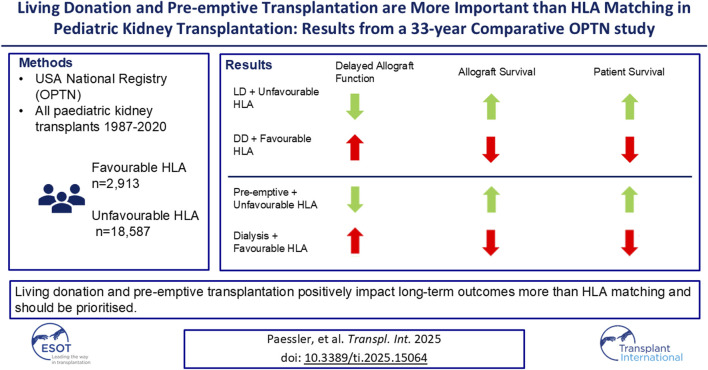

## Introduction

Due to advances in modern medicine, there is an increasing number of children with complex conditions surviving outside childhood, many with conditions affecting the kidneys. This brings up an increasing number of pediatric kidney transplant candidates. Unfortunately, organ donation rates have not increased in the same proportions. There are currently over 1,200 children waiting for a kidney transplant from a deceased donor in the United States of America (USA), however in 2023, only 548 pediatric kidney transplants took place from deceased donors (based on the Organ Procurement and Transplantation Network (OPTN) data as of November 2024).

Human Leucocyte Antigen (HLA) mismatches is one of the many variables considered when matching kidney donors and recipients [1, 2]. However, over the years its importance has diminished [3, 4]. The Share 35 policy [5], brought many changes, one of which that prioritised kidneys from deceased donors under 35 years old for pediatric recipients, and reduced the importance of HLA mismatch with the exemption of cases with 000 mismatches (no mismatches at HLA-A, -B and -DR). Furthermore, these changes were compounded in the 2014 kidney allocation scheme, which prioritized the highest-quality organs to candidates with the longest predicted survival. Some of the changes meant that that pediatric, highly sensitised and long-term dialysis patients were prioritized over other candidates regardless of HLA mismatch. This meant that for non-highly-sensitised pediatric recipients, HLA mismatch had little importance in kidney allocation [1, 6].

Patients with increasing number of HLA mismatches, have been shown to have shorter allograft survival, possible higher prevalence of post-transplant lymphoproliferative disorder (PTLD) [7] and fewer chances to have a second transplant in the future due to higher sensitisation rate [8]. These factors are particularly important to consider when transplanting children, who have a longer expected lifespan compared to adults and are more likely to require further transplants and therefore have a higher lifetime risk of being affected by potentially negative outcomes.

On the other hand, HLA matching is not the only predictor of long-term outcomes. Evidence shows that receiving transplants from living donors have superior outcomes to transplant from deceased donors [9]. Furthermore, pre-emptive transplants also lead to improved outcomes with less delayed allograft function, better allograft survival time, fewer episodes of acute rejection and a lower risk of death [10].

Due to the many possible variables that can impact outcomes for pediatric kidney transplants, it is unclear which factors should be prioritized when allocating organs. There is also emergencing evidence that other aspects of HLA matching, such as HLA-DQ or eplet mismatch load may be more important than the overall number of HLA mismatches [11–14]. However, globally, allocation systems still use HLA mismatches at HLA-A, -B and -DR in their algorithms. For many countries, particularly middle and low-income countries, the use of eplet mismatch load in day-to-day clinical practice is still a long way off and so it is important to understand HLA-A, -B and -DR better as that is the basis upon which most organs are allocated.

The primary aim of this study was to compare the outcomes of transplants with favourable and unfavourable HLA matches, and more importantly to identify whether living donation or pre-emptive transplantation contribute more significantly to allograft outcomes than HLA matching.

## Materials and Methods

The OPTN database is an online registry developed by United Network of Organ Sharing (UNOS) that contains all data pertaining to patient waiting lists, living and deceased organ donation, organ matching and organ transplants that have taken place in the U.S. since 1st October 1987 [15]. Data are added to the database at the point of listing a patient for transplant, at the point of donation and is updated at 6 months, 1-year and annually post-transplant with recipient outcome data. This database is the largest registry containing data on paediatric kidney transplants. It was chosen for this study as it would be able to provide us with the largest sample size.

OPTN registry data for all kidney transplants performed for recipients under the age of 18 years in the U.S. from October 1987 until September 2020 were requested. Data retrieved from the registry included donor and recipient demographics, number of prior transplants, dialysis status at transplantation, number of HLA mismatches at HLA-A, -B and -DR, primary allograft non-function, delayed allograft function, allograft survival and patient survival time. HLA-locus specific mismatches for each patient were not included, however mismatch level was included. HLA mismatch levels are referred to as follows:

- Mismatch level 1–000
- Mismatch level 2–0 DR and 0/1 B (100, 010, 110, 200, 210)
- Mismatch level 3–0 DR and 2B, or 1 DR and 0/1 B (020, 120, 220, 001, 101, 201, 011, 111, 211)
- Mismatch level 4–1 DR and 2B or 2 DR (021, 121, 221, 002, 102, 202, 012, 112, 212, 022, 122, 222)

All patients’ post-transplant follow up data that were used for analysis were based on their latest data submitted to the registry in January 2021. Patients with no data recorded on the level of HLA mismatches were excluded. Other missing data was assumed to be missing completely at random. For each patient we only analysed data for the first kidney transplant unless otherwise specified.

HLA Mismatch is defined in the OPTN database as occurring when the donor has at least one HLA-A, HLA-B or HLA-DR antigen that is not present in the recipient [15]. The number of HLA mismatches describes the number of antigens in the donor that are not present in the recipient and level of mismatches are described as above. This accounts for the increased immunogenicity of class 2 mismatches and accounts for the evidence that class 2 mismatches are more likely to lead to rejection than class 1 mismatches [16–18]. For the purposes of this study we defined Favourable HLA match as transplants with HLA Mismatch level 1 or 2. Unfavourable HLA matches were defined as transplants with HLA Mismatch level 3 or 4.

All statistical analysis was carried out with IBM Statistical Package for Social Sciences (SPSS) Version 28 [19]. Demographics and post-transplant outcomes including allograft and patient survival were compared between patients with different levels of HLA Mismatches. Means, standard deviations and 95% confidence intervals were reported to describe all numerical data, frequencies and percentages were used to describe categorical data. Independent T-test and Analysis of Variance (ANOVA) was used for significance testing to compare groups of patients. Patient and allograft survival at 1, 3, 5,10, 20, and 30 years post-transplant was estimated using Kaplan-Meier analysis and log-rank testing was used to assess comparisons. Multivariate cox proportional hazard regression analysis to estimate the effect of the level of HLA mismatches on allograft and patient survival while accounting for dialysis status at time of transplantation, recipient ethnicity, recipient sex, recipient age, donor type, decade of transplantation, donor creatinine and cold ischaemia time was also carried out after ruling out colinearity with variance inflation factors. Sensitivity analysis was carried out by running several different models. Hazard ratios for allograft failure and for death over time post-transplant were also presented to display how some determinants of graft failure and death are time-varying; these were presented using piece-wise exponential additive mixed models [20]. P-values, with a threshold of significance of p < 0⋅05 are displayed as a measure of significance. When data were used for multiple comparisons Bonferroni corrections were implemented.

## Results

### Demographics and Background Information

Overall, 21,500 patients met the inclusion criteria. Their distribution across the number of HLA mismatches and HLA mismatch levels can be seen in Table 1.

**TABLE 1:** Number of transplants with each number of HLA mismatches and at each HLA Mismatch Level, proportions for each number of HLA mismatches/level of HLA mismatches of total number of transplants.

Number of HLA mismatches	Number	Proportion
0	687	3.2%
1	1,107	5.2%
2	3,447	16.0%
3	5,547	25.8%
4	3,881	18.0%
5	4,515	21.0%
6	2,316	10.8%
HLA mismatch level		
1	687	3.2%
2	2,226	10.4%
3	8,634	40.2%
4	9,953	46.2%
Total	21,500	100%

The mean recipient age was 10⋅77 (10⋅63–10⋅90) years with patients with unfavourable HLA matches being significantly older – 10⋅82(10⋅75–10.89) vs. 10⋅61 (10⋅42–10⋅80) years (p < 0⋅02). Patient ethnicity, sex, underlying renal disease and era data can be seen in Table 2. White patients were more likely to have a favourable HLA match than any other ethnicity (p < 0⋅01). Over time, as the number of deceased donor transplants increased and the number of living donor transplants decreased, an increasing proportion of transplants were performed with an unfavourable HLA match (p < 0⋅01). For example, prior to 1990 21.5% of transplants had a favourable HLA match, and since 2020 this has decreased to just 8.0%.

**TABLE 2:** Number and proportions of favourable vs. unfavourable HLA matches for different ethnicities, sex, underlying renal disease and eras. Patients with no data on ethnicity, sex, renal disease or era were excluded from analysis for their respective category.

Characteristic		Favourable HLA match number	Favourable HLA match proportion	Unfavourable HLA match Number	Unfavourable HLA match proportion	p-value
Ethnicity	White	1977	67.9%	9,787	52.7%	<0.01
	Black	272	9.3%	3,644	19.6%	<0.01
	Hispanic	550	18⋅9%	4,071	21⋅9%	<0.01
	Asian	61	2⋅1%	605	3.3%	<0.01
	Other/Mixed	53	1⋅8%	480	2.6%	<0.01
Sex	Male	1718	59.0%	18,587	58⋅7%	<0.01
	Female	1,194	41.0%	7,654	41.2%	
Renal Disease	Cystic	85	7.8%	891	7⋅6%	0.79
	Obstructive/Reflux	269	24⋅8%	2,735	23⋅4%	0.29
	Glomerulo-nephritis	357	32.9%	3,819	32.7%	0.85
	Hypertension/Vascular	42	3.9%	556	4⋅8%	0.18
	Hereditary/Metabolic	56	5⋅2%	722	6⋅2%	0.18
	Hypoplasia/Dysplasia	177	16⋅3%	2,130	18.2%	0.12
	Other	98	9.0%	838	7⋅2%	0.02
Era	<1990	279	21.5%	1,018	78.5%	<0.01
	1990–1999	1,131	19.1%	4,807	80.9%	<0.01
	2000–2009	891	12.6%	6,199	87.4%	<0.01
	2010–2019	580	8.6%	6,197	91.4%	<0.01
	2020>	32	8.0%	366	92.0%	<0.01

The majority of transplants with favourable HLA matches where from living donors (77⋅0% living donors vs. 23⋅0% deceased donors), this was a significantly higher proportion than transplants with unfavourable HLA matches (13⋅8% living donors vs. 86⋅2% deceased donors) (p < 0⋅01). Patients with favourable HLA matches were more likely to have received their transplants pre-emptively than patients with unfavourable HLA matches (26⋅5% vs. 23⋅3%, p < 0⋅01).

### Post-Transplant Outcomes

Overall, transplants with favourable HLA matches were significantly less likely to have delayed allograft function (DGF) than transplants with unfavourable HLA matches (n = 204, 7.0% vs. n = 1,571, 8.5%, p < 0⋅01). However, this trend was not seen in relation to primary allograft non-function (n = 23, 0⋅8% vs. n = 194, 1⋅0%, p = 0⋅20).

**FIGURE 1:**
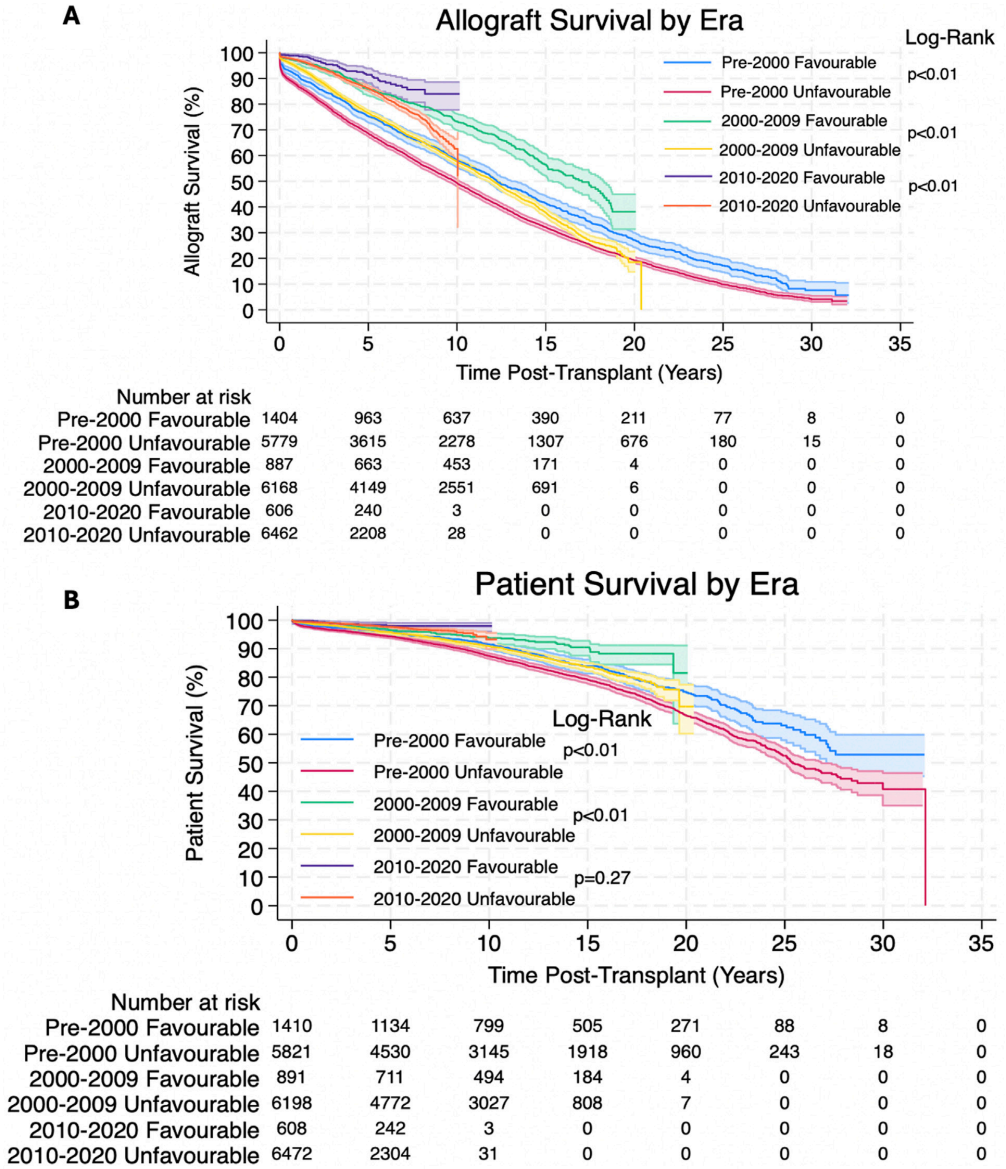
**(A,B)** Estimated Kaplan-Meier allograft and patient survival (respectively) post-transplant, stratified by transplantation era for favourable and unfavourable HLA matches. The shaded regions represent 95% confidence intervals.

Both allograft survival and patient survival were significantly better in patients with favourable HLA matches. 1, 3, 5, 10, 20 and 30 years allograft and patient survival for these patients is summarized in Table 3 and survival curves stratified by transplantation era can be seen in Figures 1A,B.

**TABLE 3:** Overall estimated Kaplan-Meier allograft and patient survival for favourable and unfavourable HLA matches at 1, 3, 5, 10, 20 and 30 years post-transplant, 95% confidence intervals given in (), p-value is the result of the Log-Rank Test.

Survival	Group	1 year	3 years	5 years	10 years	20 years	30 years	p-value
Allograft survival	Favourable HLA Match	94.1 (93.2–94.8) %	88.0 (86.8–89.1) %	80.7 (79.2–82.1) %	65.3 (63.3–67.2) %	31.7 (29.1–34.2) %	9.9 (7.1–13.2) %	**<0**.**01**
	*Number at risk*	*2,863*	*2,452*	*2,045*	*1,202*	250	16	
	Unfavourable HLA Match	93.1 (92.7–93.4) %	84.9 (84.4–85.5) %	76.6 (76.0–77.3) %	56.6 (55.7–57.5) %	21.6 (20.5–22.8) %	4.6 (3.4–6.0) %	
	*Number at risk*	*15,960*	*12,821*	*9,972*	*4,857*	682	15	
Patient survival	Favourable HLA Match	98.4 (97.9–98.8) %	97.4 (96.7–97.9) %	96.0 (95.2–96.7) %	92.5 (91.3–93.5) %	77.6 (74.8 0 89.2) %	50.7 (41.9–59.0) %	**<0**.**01**
	*Number at risk*	*2,976*	*2,651*	*2,298*	*1,442*	325	18	
	Unfavourable HLA Match	98.5 (98.3–98.7) %	97.3 (97.0–97.5) %	95.8 (95.5–96.1) %	90.1 (89.6–90.7) %	69.4 (67.8–71.1) %	42.4 (36.5–48.3) %	
	*Number at risk*	*16,785*	*14,210*	*11,606*	*6,203*	967	18	

Bold values indicate statistical significance.

**FIGURE 2:**
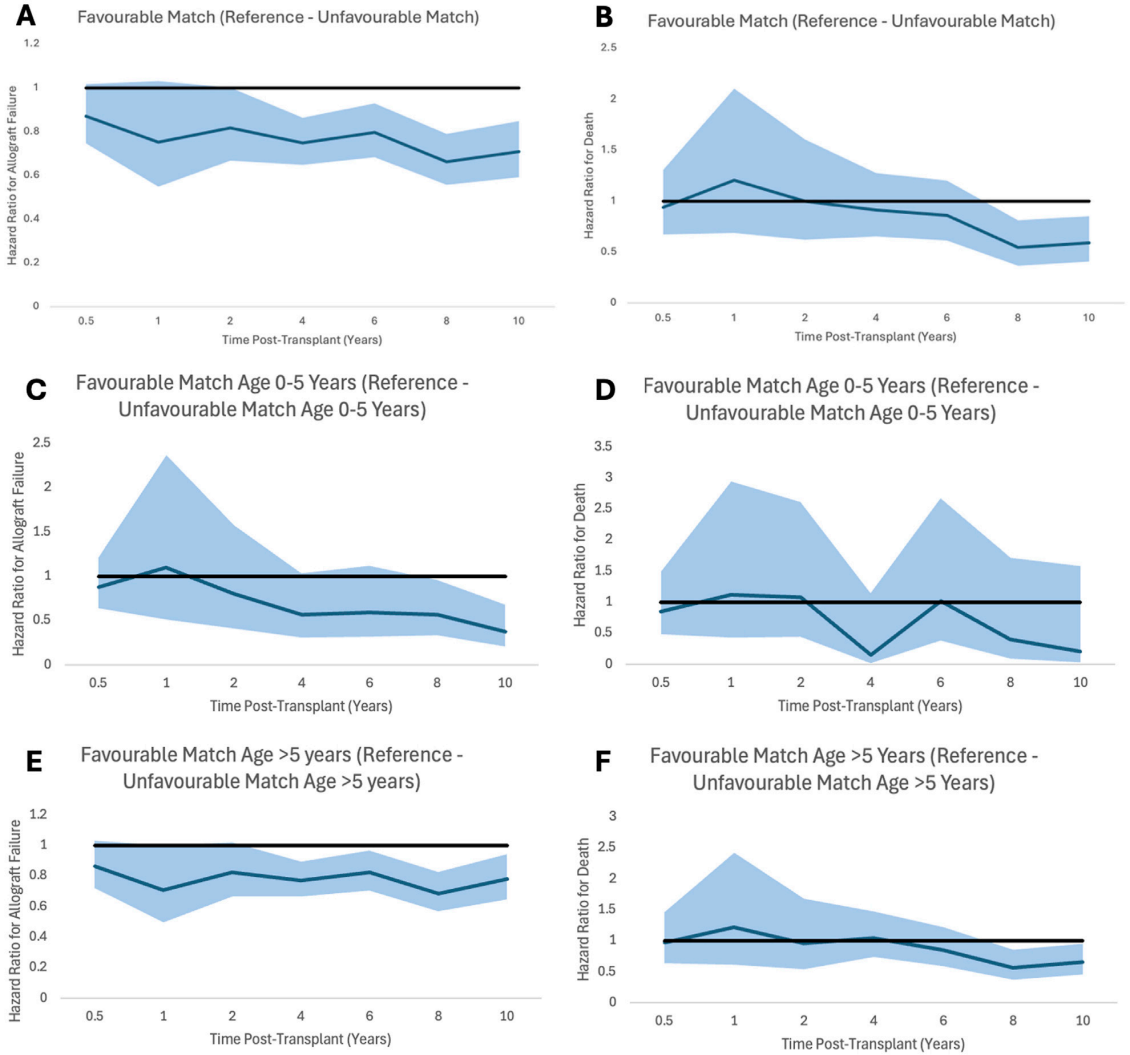
Proportional hazard ratios for allograft failure **(A,C,E)** and for death **(B,D,F)** by time post-transplant for favourable HLA matches with reference to unfavourable HLA matches generally and stratified by recipient age. Shaded areas represent 95% confidence intervals.

Multivariate Cox Proportional Hazards Regression Model of allograft failure and death was carried out to understand the impact of the HLA mismatch level, dialysis status, recipient ethnicity, recipient sex, recipient age, donor type, decade of transplantation, donor creatinine, and cold ischaemia time. Decades of transplantation are described in ascending order from <1990 to 1990–1999, 2000–2009, etc. starting with 1.00 = <1990. All the above variables have variance inflation factors of <1.15 and so colinearity was ruled out. Sensitivity analysis, Hazard ratios with 95% confidence intervals and p-values can be seen in Tables 4, 5. Graphs to show how these risks changed over time and for different age groups can be seen in Figure 2.

**TABLE 4:** Sensitivity analysis with Multivariate cox proportional hazards regression models for allograft failure for favourable and unfavourable HLA matches.

Variable	Hazard ratio	p-value	95% CI
Model 1			
Unfavourable Match	1.31	**<0.01**	1.25–1.39
Model 2			
Unfavourable Match	1.21	**<0.01**	1.07–1.38
Dialysis Prior to Transplant	1.25	**<0.01**	1.15–1.37
Non-White Recipient Ethnicity	0.98	0.05	0.96–1.00
Male	0.83	**<0.01**	0.78–0.89
Decade of Transplantation	0.74	**<0.01**	0.70–0.78
Deceased Donor	0.92	0.45	0.74–1.14
Donor Creatinine	1.02	**0.03**	1.00–1.05
Recipient Age (Years)	1.04	**<0.01**	1.04–1.06
Cold Ischaemia Time (Hours)	1.00	**<0.01**	1.00–1.01
Model 3			
Unfavourable Match	1.25	**<0.01**	1.18–1.33
Dialysis Prior to Transplant	1.24	**<0.01**	1.17–1.30
Decade of Transplantation	0.73	**<0.01**	0.71–0.75
Deceased Donor	1.37	**<0.01**	1.31–1.42
Model 4			
Unfavourable Match	1.25	**<0.01**	1.18–1.33
Dialysis Prior to Transplant	1.23	**<0.01**	1.17–1.30
Non-White Recipient Ethnicity	1.00	0.66	0.99–1.01
Decade of Transplantation	0.73	**<0.01**	0.71–0.75
Deceased Donor	1.36	**<0.01**	1.31–1.42

Bold values indicate statistical significance.

**TABLE 5:** Sensitivity analysis with Multivariate cox proportional hazards regression models for death for favourable and unfavourable HLA matches.

Variable	Hazard ratio	p-value	95% CI
Model 1			
Unfavourable Match	1.29	**<0.01**	1.15–1.43
Model 2			
Unfavourable Match	1.18	1.28	0.92–1.52
Dialysis Prior to Transplant	1.44	**<0.01**	1.20–1.75
Non-White Recipient Ethnicity	0.95	**<0.01**	0.91–0.98
Male	0.78	**<0.01**	0.69–0.89
Decade of Transplantation	0.70	**<0.01**	0.63–0.78
Deceased Donor	1.03	0.88	0.65–1.66
Donor Creatinine	1.04	0.08	0.99–1.09
Recipient Age (Years)	1.04	**<0.01**	1.03–1.07
Cold Ischaemia Time (Hours)	1.01	**0.01**	1.00–1.02
Model 3			
Unfavourable Match	1.6	**0.01**	1.03–1.30
Dialysis Prior to Transplant	1.44	**<0.01**	1.28–1.62
Decade of Transplantation	0.72	**<0.01**	0.68–0.76
Deceased Donor	1.56	**<0.01**	1.44–1.69
Model 4			
Unfavourable Match	1.16	**0.01**	1.03–1.30
Dialysis Prior to Transplant	1.45	**<0.01**	1.29–1.63
Non-White Recipient Ethnicity	0.97	**<0.01**	0.94–0.99
Decade of Transplantation	0.72	**<0.01**	0.69–0.76
Deceased Donor	1.58	**<0.01**	1.45–1.72

Bold values indicate statistical significance.

### Impact of Donor Type

Results were also compared between patients who received favourable HLA matched allografts from deceased donors and those who received unfavourable HLA matched grafts from living donors.

Transplants from living donors with unfavourable HLA matches were significantly less likely to have DGF than transplants from deceased donors with favourable HLA matches (n = 396, 5.0% vs. n = 154, 16.7%, p < 0⋅01). However, this trend was not seen in relation to primary allograft non-function (n = 69, 0⋅9% vs. n = 10, 1⋅1%, p = 0⋅49).

**FIGURE 3:**
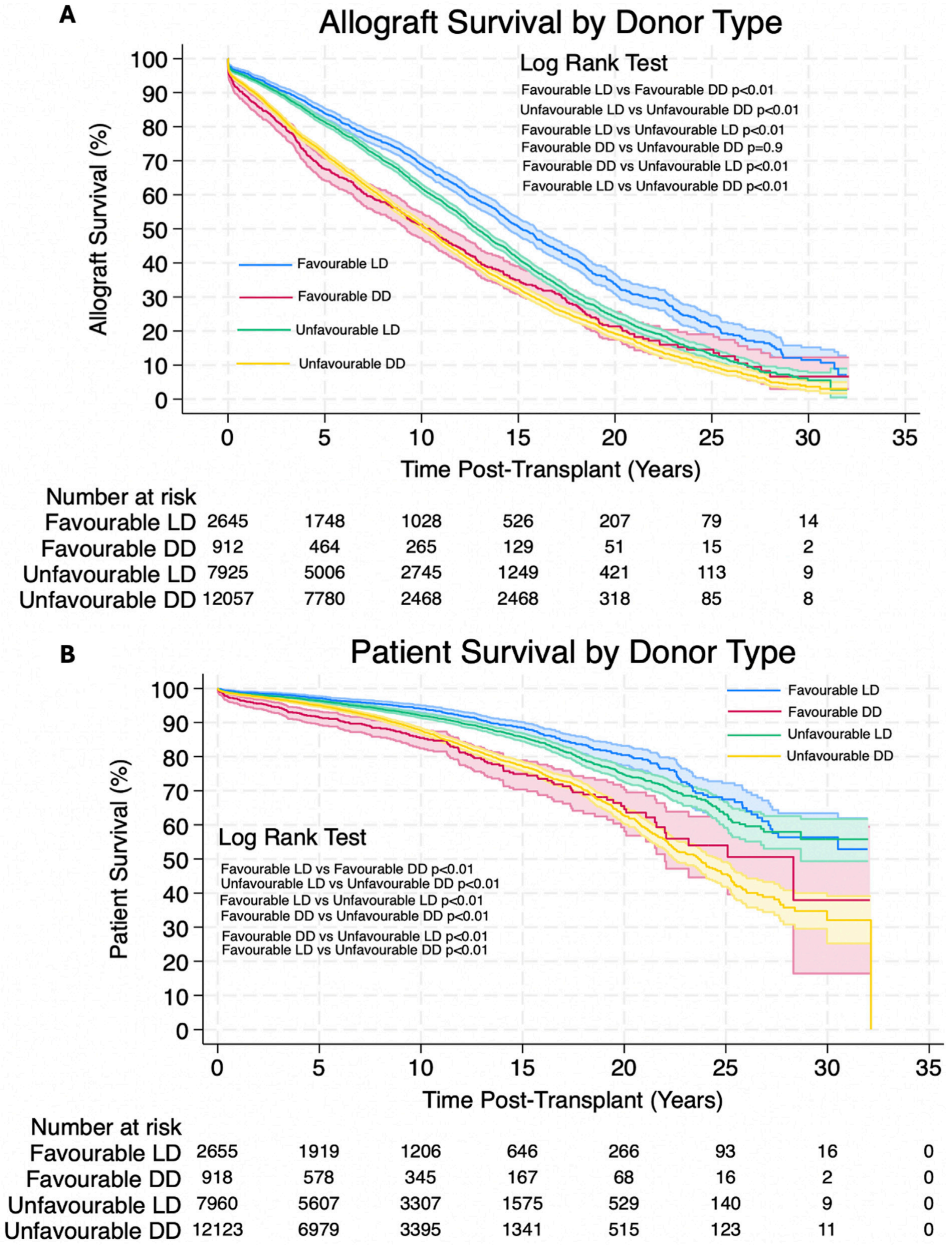
**(A,B)** Estimated Kaplan-Meier allograft and patient survival (respectively) post-transplant for favourable and unfavourable HLA matches by donor type. Shaded areas represent 95% confidence intervals.

**FIGURE 4:**
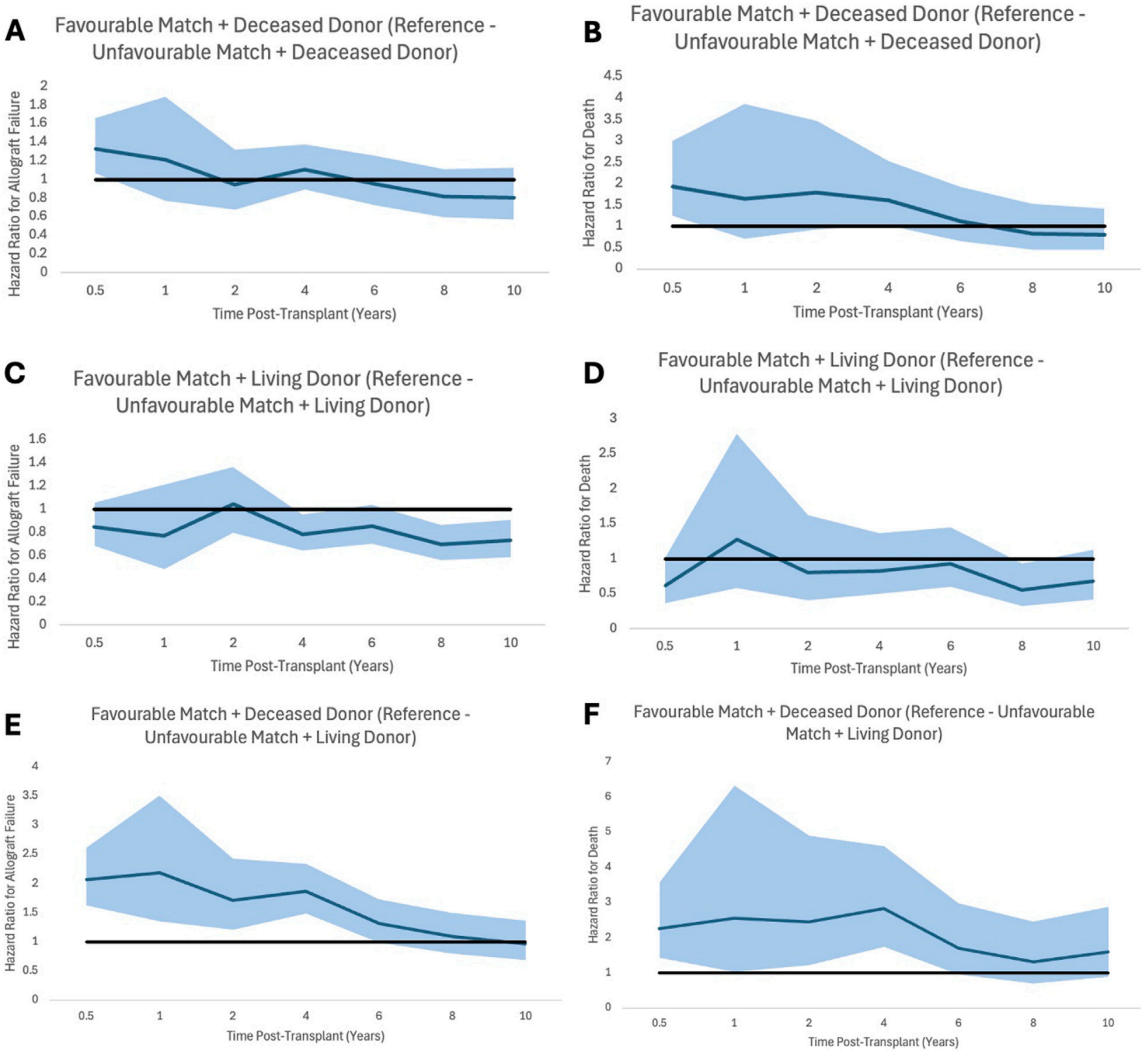
Proportional hazard ratios for allograft failure **(A,C,E)** and for death **(B,D,F)** by time post-transplant for favourable HLA matches with reference to unfavourable HLA, stratifying for donor type. Shaded areas represent 95% confidence intervals.

Analysis showed that patients receiving allografts with unfavourable HLA matches from living donors had significantly better allograft and patient survival. 1, 3, 5, 10, 20 and 30 years allograft and patient survival for these patients is summarized in Table 6 and survival curves can be seen in Figures 3A,B. Graphs to show proportional hazard ratios for allograft failure and death over time by donor type can be seen in Figure 4.

**TABLE 6:** Estimated Kaplan-Meier allograft and patient survival for patients receiving transplants with favourable HLA matches from deceased donors and unfavourable HLA matches from living donors at 1, 3, 5, 10, 20 and 30 years post-transplant, 95% confidence intervals given in (), p-value is the result of the Log-Rank Test.

Survival	Group	1 year	3 years	5 years	10 years	20 years	30 years	p-value
Allograft survival	Favourable HLA Match – Deceased Donor	88.8 (86.6–90.7) %	79.4 (76.5–82.0) %	67.5 (64.2–70.7) %	51.2 (47.4–55.0) %	21.4 (17.4–25.6) %	6.6 (3.0–12.3) %	**<0**.**01**
	*Number at risk*	*758*	*614*	*464*	*265*	51	2	
	Unfavourable HLA Match – Living Donor	94.7 (94.2–95.2) %	88.7 (88.0–89.4) %	81.4 (80.5–82.3) %	62.0 (60.7–63.2) %	24.1 (22.5–25.7) %	5.5 (3.7–7.8) %	
	*Number at risk*	*7,098*	*6,086*	*5,006*	*2,745*	421	9	
Patient survival	Favourable HLA Match – Deceased Donor	96.8 (95.4–97.7) %	94.5 (92.8–95.9) %	91.6 (89.4–93.3) %	85.5 (82.4–88.0) %	65.5 (59.3–71.1) %	37.9 (16.5–59.5)	**<0**.**01**
	*Number at risk*	*821*	*712*	*578*	*345*	68	2	
	Unfavourable HLA Match – Living Donor	98.6 (98.3–98.8) %	97.6 (97.3–97.9) %	96.4 (95.9–96.8) %	92 (91.3–92.7) %	74.8 (72.6–76.8) %	55.8 (49.4–61.7) %	
	*Number at risk*	*7,362*	*6,521*	*5,607*	*3,307*	529	9	

Bold values indicate statistical significance.

### Impact of Pre-Emptive Transplantation

Results were also compared between patients who were transplanted after a period of being on dialysis with favourable HLA matched grafts and those who were transplanted pre-emptively with unfavourable HLA matched grafts.

Pre-emptive transplants with unfavourable HLA matches were significantly less likely to have DGF than transplants with favourable HLA matches on dialysis (n = 118, 2.6% vs. n = 245, 10.3%, p < 0⋅01). This trend was not seen in relation to primary allograft non-function (n = 33, 0⋅7% vs. n = 20, 0.8%, p = 0⋅56).

**FIGURE 5:**
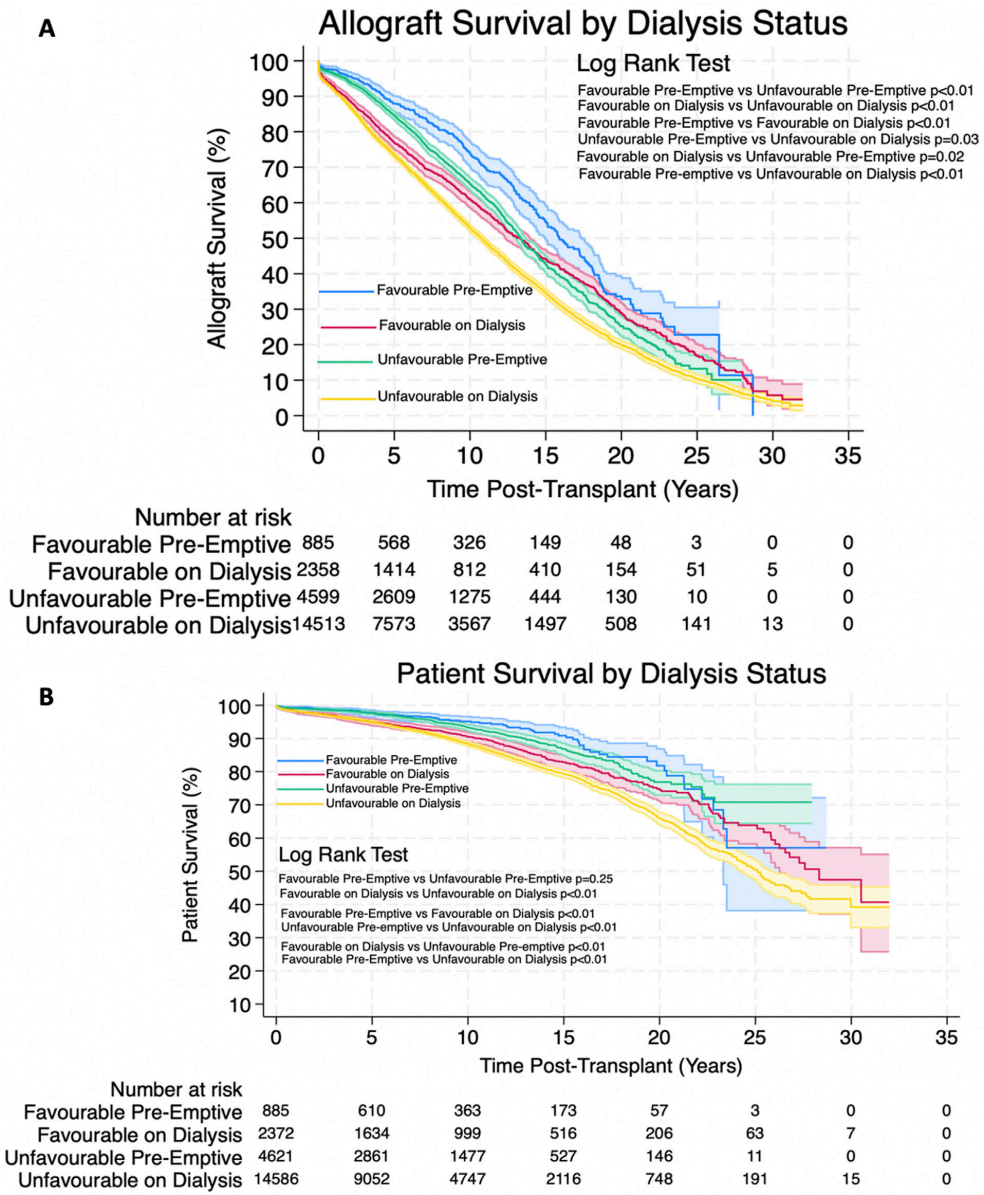
**(A,B)** Estimated Kaplan-Meier allograft and patient survival (respectively) post-transplant for favourable and unfavourable HLA matches by dialysis status prior to transplant. Shaded areas represent 95% confidence intervals.

**FIGURE 6:**
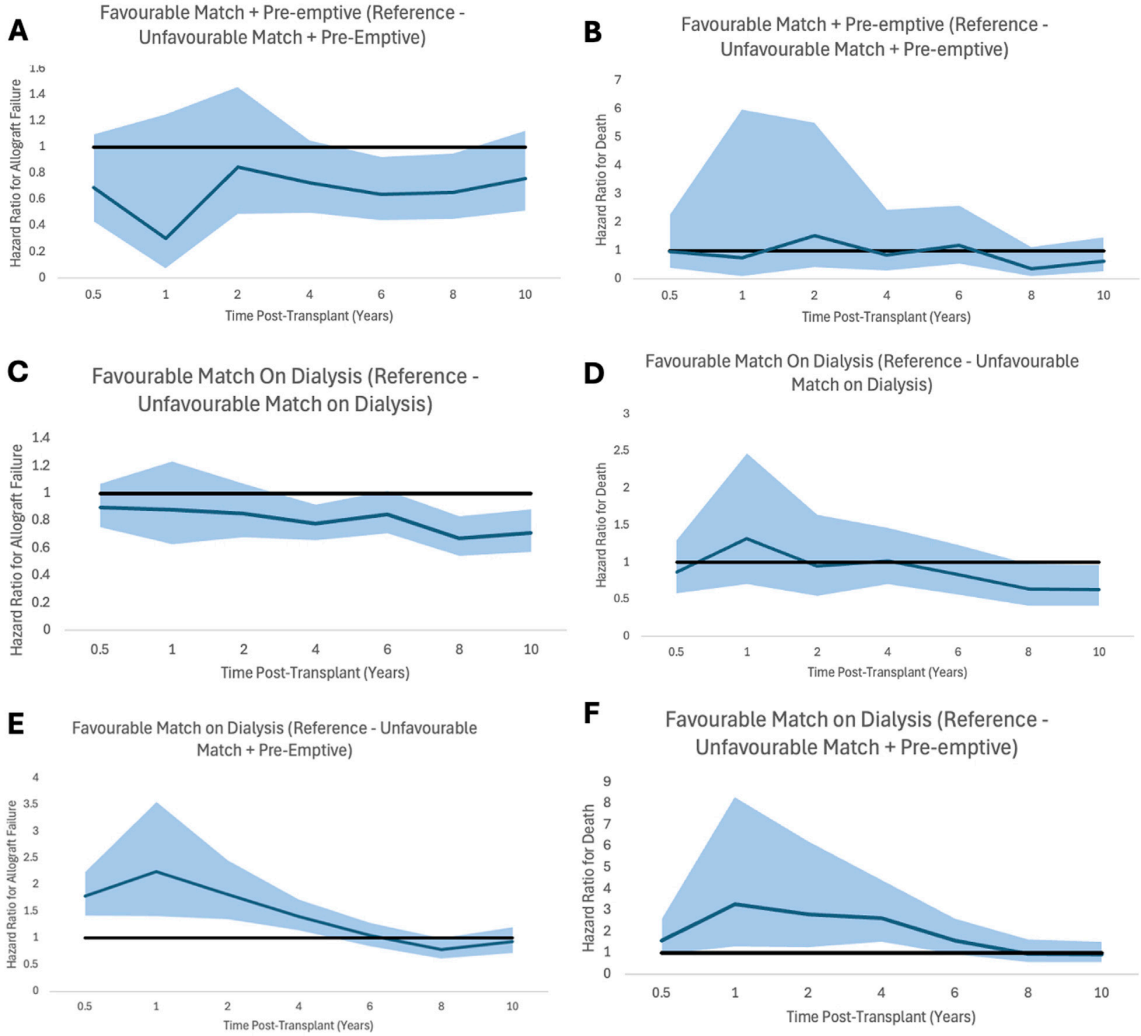
Proportional hazard ratios for allograft failure **(A,C,E)** and for death **(B,D,F)** by time post-transplant for favourable HLA matches with reference to unfavourable HLA, stratifying for dialysis status prior to transplant. Shaded areas represent 95% confidence intervals.

Analysis showed that patients receiving allografts pre-emptively with unfavourable HLA matches had significantly better allograft survival and patient survival. 1, 3, 5,10, 20 and 30 years allograft and patient survival for these patients is summarized in Table 7 and survival curves can be seen in Figures 5A,B. Graphs to show proportional hazard ratios for allograft failure and death over time by dialysis status can be seen in Figure 6.

**TABLE 7:** Estimated Kaplan-Meier allograft and patient survival for patients transplants with favourable HLA matches after a period on dialysis and pre-emptive transplants with unfavourable HLA matches at 1, 3, 5, 10, 20 and 30 years post-transplant, 95% confidence intervals given in (), p-value is the result of the Log-Rank Test.

Survival	Group	1 year	3 years	5 years	10 years	20 years	30 years	p-value
Allograft survival	Favourable HLA Match on Dialysis	92.9 (91.8–93.9) %	85.2 (83.7–86.6) %	76.9 (75.0–78.6) %	61.0 (58.7–63.3) %	28.9 (26.0–31.9) %	5.8 (2.9–9.9) %	**0.02**
	*Number at risk*	*2,069*	*1,736*	*1,414*	*812*	154	5	
	Pre-emptive Unfavourable HLA Match	96.4 (95.8%–96.9%	91.7 (90.8–92.5) %	84.6 (83.4–85.8) %	65.4 (63.6–67.1) %	25.2 (22.5–281) %	-	
	*Number at risk*	*4,087*	*3,341*	*2,609*	*1,275*	130	0	
Patient survival	Favourable HLA Match on Dialysis	98.3 (97.7–98.8) %	97.0 (96.2–97.7) %	95.0 (93.9–95.8) %	90.5 (89.0–91.9) %	74.9 (71.4–78.0) %	47.5 (37.1–57.1) %	**<0**.**01**
	*Number at risk*	*2,182*	*1,928*	*1,634*	*999*	206	7	
	Pre-emptive Unfavourable HLA Match	99.1 (98.8–99.4) %	98.6 (98.2–98.9) %	97.8 (97.3–98.2) %	93.4 (92.3–94.3) %	76.9 (73.0–80.3) %	-	
	*Number at risk*	*4,188*	*3,508*	*2,861*	*1,477*	146	0	

Bold values indicate statistical significance.

### Re-Transplantation Outcomes

Patients who had a favourable HLA match at their first transplant, were equally as likely to receive a favourable HLA match at their second transplant as those with unfavourable HLA match at their first transplant (n = 48, 16.9% vs. n = 262, 17.4%) (p = 0⋅84). Patients with a favourable HLA match at their first transplant were however more likely to receive a pre-emptive 2nd transplant compared to those with an unfavourable HLA match at their first transplant (n = 66, 23.2% vs. n = 267, 17.7%) (p = 0.03).

**FIGURE 7:**
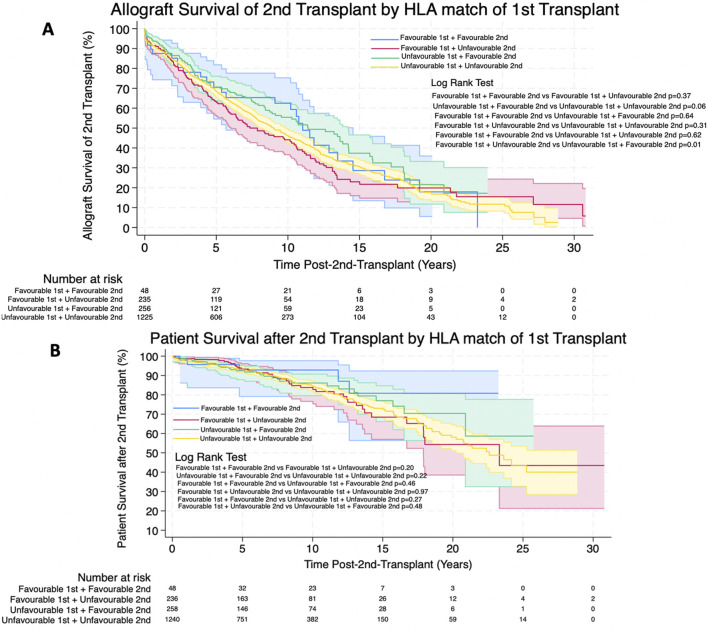
**(A,B)** Estimated Kaplan-Meier allograft and patient survival (respectively) post-re-transplant for favourable and unfavourable HLA matches by HLA match of first transplant. Shaded areas represent 95% confidence intervals.

Kaplan-Meier survival estimates for allograft survival of the 2nd allograft and patient survival after the 2nd transplant was not significantly different between patients receiving favourable and unfavourable HLA matches during their first transplant. 1, 3, 5, 10 and 20 years allograft and patient survival for these patients is summarized in Table 8 and survival curves can be seen in Figures 7A,B.

**TABLE 8:** Estimated Kaplan-Meier allograft and patient survival after re-transplantation for patients who received favourable and unfavourable HLA matches for their first transplant at 1, 3, 5,10 and 20 years post-re-transplant, 95% confidence intervals given in (), p-value is the result of the Log-Rank Test.

Survival	Group	1 year	3 years	5 years	10 years	20 years	p-value
Allograft survival of 2nd transplant	Favourable HLA Match at 1st Transplant	89.7 (85.4–92.7) %	75.5 (69.8–90.3) %	64.5 (58.3–70.1) %	47.5 (40.7–54.0) %	18.8 (12.4–26.3) %	0⋅29
	*Number at risk*	*239*	*188*	*146*	*75*	12	
	Unfavourable HLA Match at 1st Transplant	89.9 (88.2–91.3) %	79.3 (77.0–81.3) %	68.9 (66.2–71.3) %	47.7 (44.5–50.8) %	18.3 (14.7–22.1) %	
	*Number at risk*	*1,264*	*987*	*727*	*332*	48	
Patient survival after 2nd transplant	Favourable HLA Match at 1st Transplant	98.6 (96.2–99.5) %	97.8 (95.2–99.0) %	93.2 (89.2–95.8) %	85.5 (79.4–89.9) %	59.1 (45.1–70.6) %	0⋅72
	*Number at risk*	*262*	*237*	*195*	*104*	15	
	Unfavourable HLA Match at 1st Transplant	97.6 (96.7–98.3) %	95.6 (94.4–96.6) %	92.9 (91.4–94.2) %	84.8 (82.2–87.0) %	59.6 (53.2–65.5) %	
	*Number at risk*	*1,368*	*1,149*	*897*	*456*	65	

## Discussion

To date, this study describes the clinical outcomes of the largest cohort of pediatric kidney transplant recipients in the literature. Overall, the results from this study have shown that HLA matching does play an important role in both short-term and long-term outcomes, however, we identified other factors which may make a more significant impact on clinical outcomes in pediatric kidney transplantation than HLA matching. This is the first study to show that pre-emptive transplants and living donation led to improved allograft and patient survival even in those with unfavourable HLA matches, suggesting that those factors should be prioritized over HLA matching.

Patients receiving unfavourable HLA matched allografts were more likely to experience DGF, and shorter allograft and patient survival compared to patients with favourable HLA matched allografts, although these risks are more significant in later years post-transplant. This finding is in keeping with already reported data in the literature [7, 8, 21]. It may therefore be somewhat concerning that there continues to be a rise in unfavourably HLA matched transplants for children in the USA and also worldwide due to changes in the allocation policies in an attempt to improve access and reduce waiting times [6, 22]. However, it is also important to not look at the changes after new allocation policies in isolation. The introduction of both the Share35 and the 2014 kidney allocation policy has also led to an increase in young donors for pediatric recipients, an increase in pre-emptive transplantation and an improvement in the racial disparities in access to transplantation [2, 5, 6]. Although there’s been an improvement in the racial disparities, our data shows ethnic minorities continue to be more likely to receive unfavourably HLA matched allografts compared to their Caucasian peers. This could potentially be due to the fact that there is a higher prevalence of euro-caucasian HLA types in the USA and this would be reflected in the donor pool [23]. This is something that still needs to be improved upon further to provide equal access to transplant. It is however reassuring that our data showed that these disparities are not seen in long-term allograft and patient survival.

Another disparity shown in this study was the otucomes between males and females. Males were found to have a lower risk of allograft failure and death, although a reason for this was not found in this study. We suspect that as males make up a higher proportion of patients with congenital renal anomalies than females [24], and that subset of patients does not have the added risk of recurrent renal disease, that this is what may be causing that disparity, although further research is needed to explore this further.

We also found that when stratifying the risk of allograft failure and death by recipient age, the impact of an unfavourably HLA matched allograft is not as significant in younger recipients, specifically in recipients under the age of 5 years than in older recipients. We suspect that this is due to younger recipients being less immunogenic than older recipients, and less likely to amount an immune response against mismatched antigens [25, 26], although this needs to be researched further to confirm this.

Our data, in contrary to the literature, shows that receiving an unfavourable HLA matched allograft as first transplant does not decrease the likelihood of having a favourably matched graft at re-transplantation. However, it does reduce the likelihood of being transplanted pre-emptively at transplantation, which is important to consider due to the negative implications of being exposed to dialysis. One reason, that may be the case, although not demonstrated in this study, is that patients with unfavourable HLA matches at first transplant may be more highly sensitised and so may wait longer to be matched for subsequent transplants, thereby having to start dialysis [8, 18, 27]. These factors are particularly important to consider in the pediatric population who have a longer potential lifespan and so are likely to require multiple transplants in their lifetime.

While it is important to strive for favourable HLA matches in pediatric kidney transplantation, we show that it may be more important to strive for living donation and pre-emptive transplantation for pediatric recipients. Recent meta-analysis on a cohort of over 20,000 pediatric kidney transplants has shown that pre-emptive kidney transplantation leads to a lower risk of allograft loss and acute rejection [28]. Another large study found that living donation leads to significantly improved allograft survival when compared to deceased donation [9]. Studies have also shown that even HLA incompatible transplants in which recipients have a positive HLA crossmatch, are possible and can have good clinical outcomes [29]. While the literature has been able to identify multiple factors that independently improve outcomes, we have been able to show that in direct comparison to HLA matching, living donation and pre-emptive transplantation have a more significant impact and play a bigger role in long-term clinical outcomes [30, 31]. On the other hand, our data on time-varying hazards for allograft failure and death show that the protective effects of living donation and pre-emptive transplantation are more significant early on post-transplant and their protective effects diminish when approaching 10-years post-transplant.

Our multivariate cox proportional hazards regression model suggests that for unfavourable HLA mismatches the hazard ratio for allograft failure and death increased by 0.21–0.31 and 0⋅16–0⋅60 and respectively depending on which model used. However, both deceased donation and dialysis exposure led to a larger increase in hazard ratio for allograft failure and death than HLA mismatching (0.36–0.37 and 0⋅56–0.58 respectively for deceased donation, and 0.23–0.25 and 0.44–0.45 respectively for dialysis exposure). This trend was then confirmed when directly comparing favourable HLA matched allografts from deceased donors to unfavourable HLA matched allografts from living donors, as well as favourable HLA matched allografts after a period on dialysis and pre-emptive poorly HLA matched allografts. Furthermore, our data also shows that 94.3% of all deceased donor transplants had an unfavourable HLA match. So, if a patient has a potential living donor, even if they are an unfavourable HLA match, it is unlikely they will find a better match by waiting for an organ coming from a deceased donor, and in that process, one may miss the opportunity for a pre-emptive transplant.

Efforts should therefore concentrate on increasing living donation rates and transplanting pre-emptively where possible. Unfortunately living donation rates for pediatric recipients appeared to be declining in the USA. [32]. There are multiple proposed reasons for this including the kidney allocation policy changes meaning pediatric patients get priority for younger donors and shorter waiting times thereby removing some of the motivating factors for living donation [2, 5, 6, 33]. Another potential reason is the widespread adoption of more refined testing of potential living donors which could be excluding many living donors who may otherwise have been accepted in previous years [33]. There is a general decline in the health of the overall population thereby excluding a significant proportion of the population to living donation [33, 34]. The literature also suggests that the decline in living donation is more pronounced in those with lower household incomes suggesting that financial status is another key contributor [33, 35]. It is crucial to address all these factors in order to try and increase living donation rates to provide better outcomes for our patients and ensure equity in access to transplantation. Strategies that have been shown to be successful include education programs both in the home [36] and at dialysis centers [37]. An effort should also be made at reducing the barriers to living donation such as reducing the financial disincentives which should be done on a national level. Other methods such as reducing the risk of living donation with laparoscopic or robotic donor nephrectomies have also been successful at increasing living donation rates for both adult and pediatric recipients [38].

Another promising approach is the use of paired donation schemes. Evidence shows that there is no difference in the outcomes of paired and non-paired living donor kidney transplants and these may be underutilized in the USA [39] compared to other countries. Paired donation schemes are a way of increasing the donor pool and allows more patients to benefit from living donation. For example, in the UK in the previous year an additional 97 kidney transplants were carried out through the living donor kidney sharing scheme [40]. Furthermore, Spain–which has the 2^nd^ largest paired donation scheme, has shown excellent outcomes following paired donation [41]. At present, only a limited number of centers in the USA participate in a paired kidney donation programme which limits the number of paired donations that can take place [42]. Strategies to increase the number of participating centers and regions could not only improve the number of pediatric transplants that occur from living donors but can also potentially improve HLA matching between paired donors and recipients.

Encouragingly, our data shows that overall pre-emptive transplantation has increased over time, which could potentially be due to changes in allocation systems [22] but also a greater emphasis being placed on pre-emptive transplantation following the emergence of strong evidence supporting its superiority in providing longer allograft and patient survival [28]. In order to achieve the best outcomes for our patients, it is important that pre-emptive transplantation rates continue to improve.

The main strength of this study is the large sample size – this is a national study on the largest dataset within pediatric kidney transplantation that involves over 20,000 patients. This has allowed us to report on trends and identify key differences in outcomes between different groups which is crucial to advance our understanding of this cohort of patients.

However, one of the limitations of this study is that it has not considered some of the heterogeneity within HLA matching. The study spands across 3 decades across different eras of immunosuppressants and different allocation systems which could acts as a confounding factor in this study. There is also emerging evidence of some mismatch types being more significant than others and that molecular mismatches such as eplet mismatch load may play a more significant role on clinical outcomes [11–14]. While this isn’t utilized yet in clinical practice and allocation systems, it requires further research in large multi-center studies to further our understanding of HLA matching.

Furthermore, there are other variables which we were not able to control for or evaluate in this study, such as different immunosuppression regimes, different allocation policies used over time, donor age, episodes of rejection and the use of different induction agents between each centre and between different eras which will undoubtedly have also had an impact on graft outcomes.

## Conclusion

In conclusion, this to date largest study on pediatric renal transplant recipients, has shown that living donation and pre-emptive transplantation play a significant role in long-term clinical outcomes and are more significant than HLA matching when directly compared to organs coming from deceased donors, and transplants occurring after a period of dialysis. The protective effects of living donation and pre-emptive transplantation are particularly significant in the first 10 years post-transplant.

In our study, children receiving unfavourably HLA matched allografts from living donors had better allograft and patient survival compared to children receiving favourably HLA matched allografts from deceased donors. Children receiving pre-emptive transplants with an unfavourable HLA match also had lower rates of delayed allograft function as well as better allograft and patient survival when compared to children receiving non-pre-emptive transplants with a favourable HLA match. However, patients receiving unfavourably HLA matched allografts were less likely to be transplanted pre-emptively when it came to re-transplantation.

Transplanting pediatric recipients with unfavourably HLA matched living donors may be considered before consideration of being listed on the deceased donor waiting list for a better HLA match. Strategies to increase rates of living donation and pre-emptive transplantation are crucial to improving the outcomes in pediatric kidney transplant recipients. Further research is needed to fully understand the implications of HLA and HLA epitope matching on paediatric kidney transplant recipients.

## Data Availability

Publicly available datasets were analyzed in this study. This data can be found here: https://optn.transplant.hrsa.gov/data/view-data-reports/request-data/.
